# An alarmingly high nasal carriage rate of *Streptococcus pneumoniae* serotype 19F non-susceptible to multiple beta-lactam antimicrobials among Vietnamese children

**DOI:** 10.1186/s12879-019-3861-2

**Published:** 2019-03-11

**Authors:** Hien Anh Thi Nguyen, Hiroshi Fujii, Huong Thi Thu Vu, Christopher M. Parry, Anh Duc Dang, Koya Ariyoshi, Lay-Myint Yoshida

**Affiliations:** 1 National Institute of Hygiene and Epidemiology, Hanoi, Vietnam; 2 Graduate School of Biomedical Sciences, Nagasaki University, Nagasaki, Japan; 3 Liverpool School of Tropical Medicine, Liverpool, UK; 4 Department of Pediatric Infectious Diseases, Institute of Tropical Medicine, Nagasaki University, 1-12-4 Sakamoto, Nagasaki, Japan; 5 Department of Clinical Medicine, Institute of Tropical Medicine, Nagasaki University, Nagasaki, Japan

**Keywords:** *Streptococcus pneumoniae*, Pneumococcal conjugate vaccine, Antimicrobial resistance, Colonization, Community-based study

## Abstract

**Background:**

Understanding the relationship between serotype epidemiology and antimicrobial susceptibility of *Streptococcus pneumoniae* is essential for the effective introduction of pneumococcal conjugate vaccines (PCVs) and control of antimicrobial-resistant pneumococci.

**Methods:**

We conducted a community-based study in Nha Trang, central Vietnam, to clarify the serotype distribution and pattern of *S. pneumoniae* antimicrobial susceptibility in children under 5 years of age and to identify risk factors for carrying antimicrobial-resistant strains. Nasopharyngeal swabs collected from children with acute respiratory infections (ARIs) hospitalized between April 7, 2008, and March 30, 2009, and from healthy children randomly selected in July 2008 were subjected to bacterial culture. Minimum inhibitory concentrations (MICs) against *S. pneumoniae* were determined, and multiplex-polymerase chain reaction (PCR) serotyping assays were performed. Logistic regression was applied to identify risk factors.

**Results:**

We collected 883 samples from 331 healthy children and 552 ARI cases; *S. pneumoniae* was isolated from 95 (28.7%) healthy children and 202 (36.6%) ARI cases. Age and daycare attendance were significantly associated with pneumococcal carriage. In total, 18.0, 25.8 and 75.6% of the isolates had high MICs for penicillin (≥4 μg/ml), cefotaxime (≥2 μg/ml) and meropenem (≥0.5 μg/ml), respectively. The presence of pneumococci non-susceptible to multiple beta-lactams was significantly associated with serotype 19F (Odds Ratio: 4.23) and daycare attendance (Odds Ratio: 2.56) but not ARIs, age or prior antimicrobial use. The majority of isolates non-susceptible to multiple beta-lactams (90%) were PCV13 vaccine serotypes.

**Conclusions:**

*S. pneumoniae* serotype 19F isolates non-susceptible to multiple beta-lactams are widely prevalent among Vietnamese children. Vaccine introduction is expected to significantly increase drug susceptibility.

**Electronic supplementary material:**

The online version of this article (10.1186/s12879-019-3861-2) contains supplementary material, which is available to authorized users.

## Background

*Streptococcus pneumoniae* causes an enormous global disease burden, especially in developing countries [1]. Approximately 0.4 million deaths from pneumococcal pneumonia in children younger than 5 years of age are estimated to occur annually, of which approximately 80% occur in Africa and southeast Asia [1]. In the developed world, where the pneumococcal conjugate vaccine (PCV) for infants has been introduced, both the incidence of invasive pneumococcal disease (IPD) and the hospitalization rate for IPD have decreased [2, 3]. In addition to IPD, reductions in all-cause pneumonia, pneumococcal pneumonia hospitalizations and inpatient mortality have been observed [4, 5].

Several previous epidemiological studies in Vietnam have shown that antimicrobial resistance, including penicillin-resistant *S. pneumoniae* (PRSP), is quite common among both clinical and carriage isolates [6–10]. Multidrug-resistant, globally circulating clones (Spain^23F^-1 and Taiwan^19F^-14) and their related strains are primarily responsible for resistance [7, 9]. Serogroups/serotypes 19, 23, 14 and 6 are predominant in Vietnam [6, 7]. However, no previous studies using community-based surveys have demonstrated the risk of carrying antimicrobial-resistant *S. pneumoniae* among healthy children or increased resistance to a wide range of beta-lactam antimicrobials, including carbapenems.

The introduction of PCV in the USA reduced vaccine serotype non-susceptible *S. pneumoniae* carriage and disease [11, 12]. However, compensatory increases in less susceptible non-vaccine types have been subsequently observed [13, 14]. A recent double-blind study confirmed that the 13-valent pneumococcal conjugate vaccine (PCV-13) was superior to the 7-valent pneumococcal conjugate vaccine (PCV-7) in reducing the non-susceptible *S. pneumoniae* carriage [15]. Understanding the serotype distribution of colonized *S. pneumoniae* and how the distribution relates to antimicrobial susceptibility is essential for the effective introduction of PCV and the control of drug resistance.

The main objectives of the present study are to clarify the serotype distribution and pattern of antimicrobial susceptibility of *S. pneumoniae* isolates colonizing children under 5 years of age in Nha Trang, central Vietnam, and to determine the proportion of vaccine serotypes among the resistant strains.

## Methods

### Study setting

A community-based *S. pneumoniae* colonization study was conducted in Nha Trang city in central Vietnam. Nha Trang, the capital city of Khanh Hoa province, is an optimal geographic location for population-based surveillance because the eastern side of the city is located on the sea, and the other borders are surrounded by mountains [16].

Nasopharyngeal swab samples were consecutively collected from children under 5 years of age with acute respiratory infections (ARIs) who were admitted to the pediatric ward at Khanh Hoa General Hospital between April 7, 2008, and March 30, 2009. This hospital has a capacity of 1000 beds and is the only hospital providing in-patient care in the city. Nasopharyngeal samples were collected at the time of hospital admission before starting antimicrobial treatment in the pediatric ward. The inclusion criteria for these ARI cases were described elsewhere [17]. To obtain population-representative nasopharyngeal swab samples, we also recruited healthy children under 5 years of age from two of the 16 communes in the study catchment area in July 2008 using census data [18]. The inclusion criteria for the healthy children were a lack of fever, signs of respiratory infections or history of taking antimicrobials within 1 month prior to enrollment. The caregivers provided written informed consent. Information about potential risk factors was gathered using questionnaires (the questionnaire for data collection was submitted as Additional file 1: Figure S1).

Nasopharyngeal swab samples were collected following the standardized methods provided by the WHO working group [19]. Each normal saline (1 ml)-suspended sample was divided into two aliquots. One aliquot was used for bacterial culture and antimicrobial susceptibility testing. The second aliquot was processed using molecular techniques, including identification of pneumococcus by *lytA* or *cpsA* polymerase chain reaction (PCR) and molecular serotyping. A PCR assay targeting *lytA* was done as part of a multiplex PCR to detect three major respiratory pathogens (*S. pneumoniae*, *Haemophilus influenaze* and *Moraxella catarrhalis*). The PCR was performed in 25 μl volume; each reaction tube contained 5 × PCR buffer, 200 μM of deoxynucleoside triphosphate, 1.5 mM of MgCl_2_, 1.0 U of Go Taq DNA polymerase (Promega, Madison, WI) and 0 .5μM primers. 6 μl of crude extract was used as the DNA template. Thermal cycling was performed under the following condition: 94 °C for 5 min followed by 35 cycles of 94 °C for 30 s, 55 °C for 30 s, and 72 °C for 45 s. PCR assays targeting *cpsA* were done as part of 9 multiplex PCR assays for molecular serotyping. The first reaction contained primers for serotypes 14, 6, 19F, 23F, 11A, and the universal capsular primers (*cpsA*-F and *cpsA*-R). This was followed by the second reaction for serotypes 10F/10C/33C, 34, 15B/C, 19A, and 23A. 3 μl of DNA template was used. Thermal cycling was performed under the following condition: 94 °C for 3 min followed by 30 cycles of 94 °C for 45 s, 54 °C for 45 s, and 72 °C for 60 s. The primers used for the *lytA* and *cpsA* assays were *lytA*-F 5′-TCGTGCGTTTTAATTCCAGC, *lytA*-R 5′-TGAGGGACTACCGCCTTTAT, *cpsA*-F 5′-GCAGTACAGCAGTTTGTTGGACTGACC and *cpsA*-R 5′-GAATATTTTCATTATCAGTCCCAGTC. Details of the sample treatment were described elsewhere [20].

### Susceptibility testing [21]

Nasopharyngeal swab samples were subjected to bacterial culture on site. A bacterial culture was performed on trypticase soy agar (Becton Dickinson and Company, Sparks, NV) with 7% defibrinated rabbit blood. Serial dilution (1:10^2^ to 10^7^) was performed before streaking to obtain single colonies. The plates were incubated in CO_2_ incubators at the microbiology laboratory at the study hospital. Potential *S. pneumoniae* colonies were selected based on colony morphology (alpha-hemolytic soft colonies with a central dimple [22]) and confirmed by optochin susceptibility. A bile solubility test was used if the optochin test was inconclusive.

Susceptibility testing was performed subsequently in a research laboratory. The minimum inhibitory concentrations (MICs) of 20 antimicrobial agents against *S. pneumoniae* isolates were determined using the agar dilution method with Mueller-Hinton agar (Becton Dickinson and Company, Sparks, NV) supplemented with 5% defibrinated horse blood. The antimicrobials used in the present study were penicillin (PEN) (Meiji Seika, Tokyo, Japan), amoxicillin (Sigma-Aldrich, St. Louis, MO), ampicillin, amoxicillin/clavulanate (Wako, Osaka, Japan), cefaclor (Shionogi, Osaka, Japan), cefuroxime (Shin Nihon Jitsugyo, Tokyo, Japan), cefotaxime (CTX) (Sigma-Aldrich, St. Louis, MO), cefepime (U.S. Pharmacopeia, Rockville, MD), imipenem, meropenem (MEM), erythromycin (Wako, Osaka, Japan), azithromycin (LKT Laboratories, St. Paul, MN), clarithromycin (Taisho Pharmaceutical, Tokyo, Japan), clindamycin, tetracycline, chloramphenicol (Wako, Osaka, Japan), trimethoprim/sulfamethoxazole (Sigma-Aldrich, St. Louis, MO), ofloxacin, rifampicin (Daiichi Pharmaceutical, Tokyo, Japan), and vancomycin (Shionogi, Osaka, Japan). For PEN and CTX, doubling dilutions over the concentration range of 0.004 to 128 μg/ml. For the other antimicrobials, appropriate breakpoint concentrations were used. The results were interpreted according to the breakpoints of the Clinical and Laboratory Standards Institute (CLSI) criteria (2016) [23]. *S. pneumoniae* ATCC® 49619 was used as the quality control strain.

### Serotyping

A multiplex-PCR method that was described elsewhere [24, 25] was applied to confirm the pneumococcal serotypes. A non-typeable (NT) status was assigned for *lytA*-positive samples that were *cpsA* negative.

### Genotyping of antimicrobial resistance genes

To identify PEN and other beta-lactam resistance genes in the pneumococcal culture-positive samples, we performed PCR with three primer sets (*pbp1A*, *pbp2b* and *pbp2x*) designed for PEN susceptible strains (Wakunaga Pharmaceutical, Osaka, Japan) according to the manufacturer’s instructions [26]. Macrolide-resistant genes (*mefA* and *ermB*) were also amplified [27].

### Analysis

*S. pneumoniae* carriage was defined as a nasopharyngeal sample that was culture positive for *S. pneumoniae* and *lytA* positive, or culture positive for *S. pneumoniae* and *cpsA* positive. Logistic regression analyses were performed to identify risk factors for pneumococcal carriage and carriage of non-susceptible isolates.

## Results

### Pneumococcal carriage

In total, 883 nasopharyngeal samples were collected from 552 ARI cases and 331 healthy children. Potential *S. pneumoniae* isolates were obtained in 343 cases (223 ARI cases and 120 healthy children). Of those cases, *lytA* and *cpsA* positive results were observed in 266 (77.6%) and 277 (80.8%), respectively. In 31 cases (9.0%), *lytA* positive results were not obtained but *cpsA* genes were amplified; consequently, *S. pneumoniae* carriage was confirmed by the detection of the *lytA* or *cpsA* genes in 297 cases (33.6%, 95% confidence interval (CI) 30.6–36.8). Of those cases, 202 (36.6%, 32.7–40.7) were ARI cases, and 95 (28.7%, 24.1–33.8) were healthy children.

Table 1 shows the baseline characteristics of children with and without *S. pneumoniae* carriage and the risk factors for carriage. *S. pneumoniae* carriage varied significantly by age group; carriage was significantly higher in ARI cases than in healthy children in the 1–2 yr. and ≥ 3 yr. age groups but not among infants (6–11 m) (Additional file 2: Figure S2). In the multiple logistic regression analysis, the age group and daycare attendance were significantly associated with *S. pneumoniae* carriage. Although fluctuation of the carriage rate was observed throughout the year [28], the carriage rates were not significantly different between the four periods (Apr-Jun 2008, Jul-Sep 2008, Oct-Dec 2008 and Jan-Mar 2009) among children with ARIs in this study. The carriage rates were also not significantly different between 3 months (Apr 2008, Mar 2009 and Jul 2009). Consequently, we performed analyses using data obtained from healthy children in July 2008 and ARI cases throughout the entire year.

**Table 1:** Univariate and Multiple Logistic Regression Analyses of *S. pneumoniae* Detection in the Nasopharynx by Explanatory Variables

Variables	Pneumococcal carriage (+)	Pneumococcal carriage (−)	Subtotal	Odds ratio (95% CI) (univariate)	P	Odds ratio (95% CI) (multiple)	P
Gender							
Male	161 (34.1%)	311	472	0.92 (0.69–1.23)	0.5874	0.85 (0.63–1.14)	0.2725
Female	124 (35.9%)	221	345				
Age group							
6–11 m	74 (39.8%)	112	186	Reference		0.63 (0.43–0.93)*	0.0203
1–2 yr	175 (37.1%)	297	472	0.89 (0.63–1.26)	0.5189		
≥ 3 yr	36 (22.6%)	123	159	0.44 (0.28–0.71)	0.0007		
Daycare attendance							
Yes	156 (37.9%)	256	412	1.30 (0.98–1.74)	0.0714	1.56 (1.13–2.17)	0.0073
No	129 (31.9%)	276	405				
Situation							
ARI	190 (39.1%)	296	486	1.59 (1.18–2.15)	0.0022	1.33 (0.93–1.90)	0.1137
Healthy children	95 (28.7%)	236	331				
Living in a large family (≥ 5 persons)							
Yes	153 (34.9%)	286	439	1.00 (0.75–1.33)	0.9836		
No	132 (34.9%)	246	378				
Prior antibiotic use							
Yes	100 (43.7%)	129	229	1.69 (1.23–2.31)	0.0010	1.42 (0.98–2.05)	0.0621
No or unknown	185 (31.5%)	403	588				
Season							
Apr-Jun 2008	41 (38.3%)	66	107	Reference			
Jul-Sept 2008	142 (31.8%)	305	447	0.75 (0.48–1.16)	0.1956		
Oct-Dec 2008	54 (41.2%)	77	131	1.13 (0.67–1.90)	0.6491		
Jan-Mar 2009	48 (36.4%)	84	132	0.92 (0.54–1.56)	0.7560		

This analysis was performed after exclusion of ARI cases in children under 6 m of age. *: ≥ 1 yr. vs < 1 yr

### Antimicrobial susceptibility testing

The susceptibility patterns and the MIC_50_ and MIC_90_ values against the *S. pneumoniae* isolates from the study participants for 20 antimicrobials were interpreted according to the CLSI breakpoints (Table 2, Additional file 3: Table S1, and Additional file 4: Table S2). Alarmingly high MIC values for multiple beta-lactam antimicrobials, including carbapenems, were found among the *S. pneumoniae* isolates from both the ARI cases and healthy children. The percentages of *S. pneumoniae* isolates non-susceptible to PEN (non-meningitis breakpoint: ≥4 μg/ml), CTX (non-meningitis breakpoint: ≥2 μg/ml) and MEM (≥0.5 μg/ml) were 18.0, 25.8 and 75.6%, respectively. A high rate of non-susceptibility to the other antibiotic groups was also observed. *S. pneumoniae* isolates fully resistant to three or more antibiotic groups (multidrug-resistant) accounted for 94.9% of the total isolates if resistance to PEN was defined as an MIC ≥8 μg/ml.

**Table 2:** Susceptibility of *S. pneumoniae* Isolates to Antimicrobial Agents

	Susceptible	Non-susceptible
Penicillin parenteral (non-meningitis)	242 (82.0%)	53 (18.0%)
Penicillin parenteral (meningitis)	10 (3.4%)	285 (96.6%)
Penicillin (oral penicillin)	10 (3.4%)	285 (96.6%)
Amoxicillin (non-meningitis)	226 (76.6%)	69 (23.4%)
Amoxicillin-clavulanic acid (non-meningitis)	258 (87.5%)	37 (12.5%)
Cefaclor	38 (12.9%)	257 (87.1%)
Cefuroxime (oral)	90 (30.5%)	205 (69.5%)
Cefotaxime (non-meningitis)	219 (74.2%)	76 (25.8%)
Cefotaxime (meningitis)	139 (47.1%)	156 (52.9%)
Cefepime (non-meningitis)	220 (74.6%)	75 (25.4%)
Cefepime (meningitis)	100 (33.9%)	195 (66.1%)
Imipenem	85 (28.8%)	210 (71.2%)
Meropenem	72 (24.4%)	223 (75.6%)
Erythromycin	38 (12.9%)	257 (87.1%)
Azithromycin	32 (10.8%)	263 (89.2%)
Clarithromycin	41 (13.9%)	254 (86.1%)
Clindamycin	52 (17.6%)	243 (82.4%)
Tetracycline	32 (10.8%)	263 (89.2%)
Chloramphenicol	166 (56.3%)	129 (43.7%)
Trimethoprim-sulfamethoxazole	26 (8.8%)	269 (91.2%)
Ofloxacin	226 (76.6%)	69 (23.4%)
Rifampicin	293 (99.3%)	2 (0.7%)
Vancomycin	295 (100%)	–

To confirm our results of antimicrobial susceptibility using the agar dilution method, 43 samples were selected from our study, and the microdilution method was performed according to the CLSI guidelines [23, 29]. The percentages of *S. pneumoniae* isolates non-susceptible to PEN, CTX and MEM among the 43 isolates were 30.2% vs 32.6, 41.9% vs 62.8 and 86.0% vs 86.0% (agar dilution vs microdilution), respectively.

### Risk of carrying isolates non-susceptible to multiple beta-lactams

A considerable number of the isolates had high MICs against multiple beta-lactam antimicrobials. An isolate that was non-susceptible to multiple beta-lactam antimicrobials was defined as an isolate with a PEN MIC ≥4 μg/ml, CTX MIC ≥2 μg/ml and MEM MIC ≥0.5 μg/ml; these isolates were further analyzed. These isolates accounted for 13.6% (95% CI: 10.1–17.9) of all isolates, and almost all of the isolates (38/40 isolates, 95.0%) were also non-susceptible to all of the macrolides (erythromycin, clarithromycin, and azithromycin), clindamycin, tetracycline and trimethoprim/sulfamethoxazole. We analyzed the risk or relevant factors for carriage of isolates non-susceptible to multiple beta-lactam antimicrobials (Table 3) and found that daycare attendance was the only significant risk factor. None of the other factors, including ARI hospitalization, age group, or prior antimicrobial use, were significantly associated.

**Table 3:** Univariate and Multiple Logistic Regression Risk Analyses of the Presence of Multiple Beta-lactam Non-Susceptible Isolates* by Explanatory Variables

Variables	Multiple beta-lactam non-susceptible isolates (*n* = 40)	Other pneumococcal isolates (*n* = 234)	Subtotal	Odds ratio (95% CI) (univariate)	P	Odds ratio (95% CI) (multiple)	P
Gender							
Male	22 (14.2%)	133 (85.8%)	155 (100%)	Reference			
Female	18 (15.1%)	101 (84.9%)	119 (100%)	1.08 (0.55–2.11)	0.8285		
Age group							
6–11 m	11 (15.3%)	61 (84.7%)	72 (100%)	Reference			
1–2 yr	26 (15.5%)	142 (84.5%)	168 (100%)	1.02 (0.47–2.18)	0.9689		
≥ 3 yr	3 (8.8%)	31 (91.2%)	34 (100%)	0.54 (0.14–2.07)	0.3596		
Daycare attendance							
Yes	29 (19.2%)	122 (80.8%)	151 (100%)	2.42 (1.15–5.07)	0.0167	2.56 (1.22–5.73)	0.0123
No	11 (8.9%)	112 (91.1%)	123 (100%)	Reference			
Situation							
ARI	18 (9.9%)	164 (90.1%)	182 (100%)	0.35 (0.18–0.69)	0.0019	0.48 (0.20–1.08)	0.0764
Healthy children	22 (23.9%)	70 (76.1%)	92 (100%)	Reference			
Living in a large family (≥ 5 persons)							
Yes	26 (17.7%)	121 (82.3%)	147 (100%)	1.73 (0.86–3.49)	0.1193	1.72 (0.84–3.65)	0.1423
No	14 (11.0%)	113 (88.9%)	127 (100%)	Reference			
Prior antibiotic use							
Yes	8 (8.3%)	89 (91.8%)	97 (100%)	0.41 (0.18–0.92)	0.0275	0.65 (0.23–1.76)	0.3975
No or unknown	32 (18.1%)	145 (81.9%)	177 (100%)	Reference			

This analysis was performed for cases with pneumococcal isolates that contained a single serotype (*n* = 274) after exclusion of ARI cases in children < 6 m

*PEN MIC ≥4 μg/mL, CTX MIC ≥2 μg/mL and MEM MIC ≥0.5 μg/mL

### Serotyping

Serotyping results were available for 92 healthy children and 202 ARI cases. Serogroup 6 was most common in both groups, followed by 19F (Additional file 5: Figure S3). The PCV-13 serotypes accounted for 80.4 and 72.3% of the isolates from the healthy children and ARI cases, respectively.

**Fig. 1:**
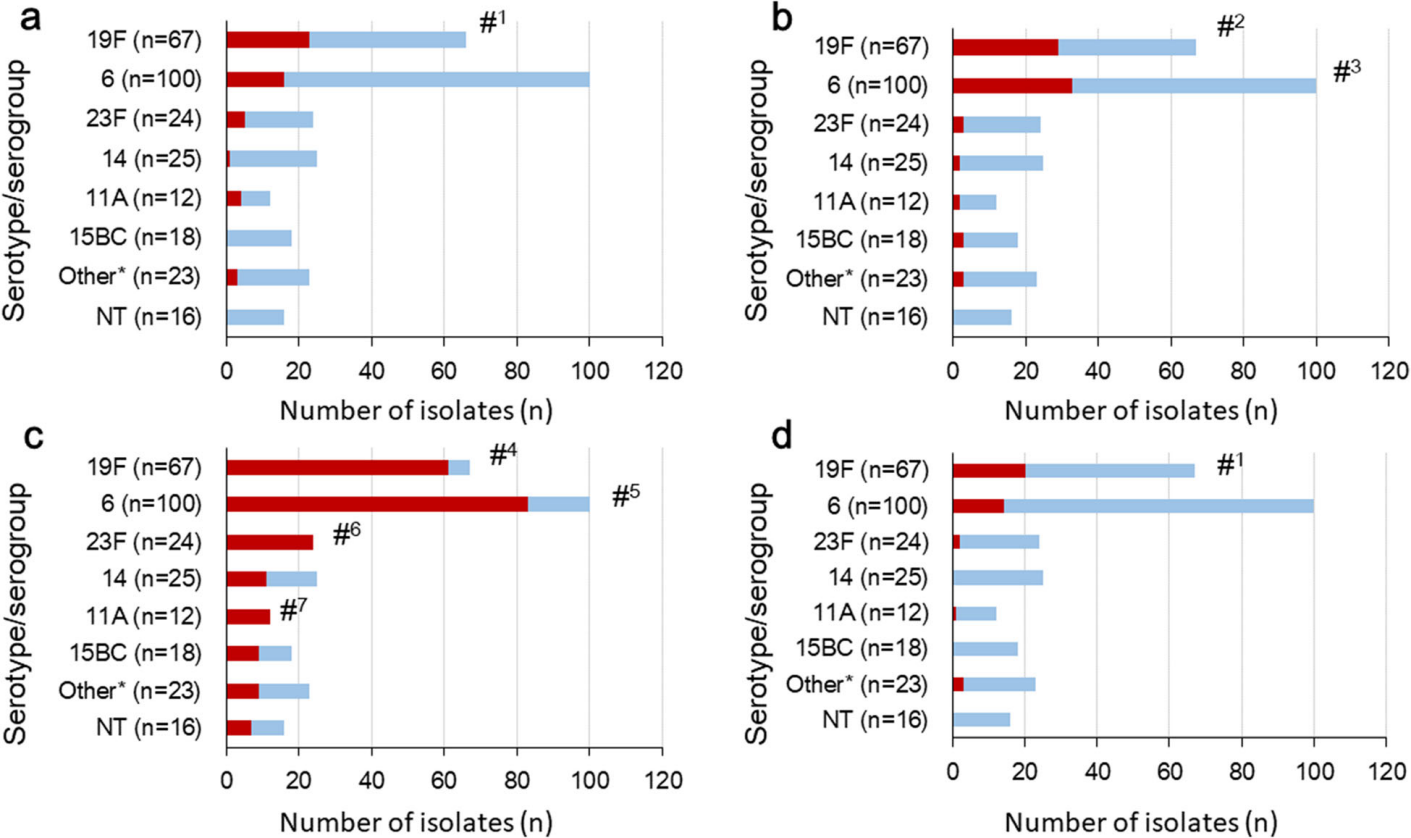
The proportions of *S. pneumoniae* isolates non-susceptible to beta-lactam antimicrobials by serotypes/serogroups (**a**: PEN, **b**: CTX, **c**: MEM, **d**: multiple beta-lactam antimicrobials). Non-susceptible isolates (PEN MIC ≥4 μg/ml, CTX MIC ≥2 μg/ml, MEM MIC ≥0.5 μg/ml and multiple beta-lactams) are shown in red. *: includes serotypes 23A, 29, and 34. The analysis was performed using pneumococcal isolates with a single serotype by multiplex-PCR (*n* = 285). #^1^: *p* < 0.0001, #^2^: *p* = 0.0002, #^3^: *p* = 0.0468, #^4^: *p* = 0.0005, #^5^: *p* = 0.0187, #^6^: *p* = 0.0029, #^7^: *p* = 0.0397

The proportions of *S. pneumoniae* isolates non-susceptible to PEN, CTX, MEM and all these three beta-lactam antimicrobials by serotypes/serogroups are shown in Fig. 1a-d and Additional file 6: Table S3. Serotype 19F was significantly associated with the carriage of *S. pneumoniae* non-susceptible to PEN (*p* < 0.0001), CTX (*p* = 0.0002), MEM (*p* = 0.0005) and all three beta-lactam antimicrobials (p < 0.0001), and serogroup 6 was significantly associated with the carriage of *S. pneumoniae* non-susceptible to CTX (*p* = 0.0468) and MEM (*p* = 0.0187). The detection of pneumococcal isolates non-susceptible to MEM was also significantly associated with serotypes 23F (*p* = 0.0029) and 11A (*p* = 0.0397). Of the *S. pneumoniae* isolates non-susceptible to all three beta-lactam antimicrobials, the majority (90.0%) were of the PCV13-vaccine serotype.

### Genotyping of antimicrobial resistance genes

The majority of the *S. pneumoniae*-positive samples (265/290, 91.4%: 95% CI 87.6–94.1) were also positive for genotypic penicillin-resistant *S. pneumoniae* (*gPRSP*) (*pbp1a* + *2x* + *2b*). The genotypic penicillin-sensitive *S. pneumoniae* (*gPSSP*) samples accounted for only 2.4% (95% CI: 1.17–4.90) of the isolates. A total of 282 samples (97.2%: 95% CI 94.7–98.6) were positive for either *ermB* or *mefA*. Details are shown in Additional file 7: Table S4 and Additional file 8: Table S5.

### The impact of pneumococci non-susceptible to multiple beta-lactam antimicrobials on clinical indices

Clinical indices were evaluated for ARI cases where pneumococci were detected (*n* = 202). The detection of pneumococci non-susceptible to multiple beta-lactam antimicrobials (*n* = 18) among the ARI cases did not lead to significant prolongation of hospitalization and did not affect the type of antimicrobials administered during the hospital stay.

## Discussion

This study is the first to report that an alarmingly high proportion of children in Vietnam carry *S. pneumoniae* isolates non-susceptible to multiple beta-lactam antimicrobials, including carbapenems. These high levels of non-susceptibility were observed regardless of whether the child was sick with an ARI in the hospital or healthy in the community. These results were obtained using a population-based study at a site in central Vietnam. The proportions of non-susceptible pneumococcal isolates with high MICs (cefotaxime or ceftriaxone MIC ≥2 μg/ml) were 0–4.4% in previous studies conducted in Vietnam, even among clinical isolates [6–10]. Although a few studies demonstrated significant increases in pneumococci non-susceptible to cefotaxime among nasopharyngeal carriage isolates, the target populations of these studies were daycare attendees or children who visited a clinic (including health check visits) [30–32], who were not necessarily representative of healthy children living in the community. The present study clearly showed that *S. pneumoniae* strains non-susceptible to multiple beta-lactam antimicrobials were circulating deep in the community of central Vietnam.

The proportion of pneumococcal isolates non-susceptible to multiple beta-lactam antimicrobials was not affected by prior antimicrobial use. The association between resistant pneumococci (both colonization and clinical samples) and the area of consumption of antimicrobials has been described in an ecological study [33], a cross-sectional study [34] and a cluster-randomized trial [35], suggesting the importance of antibiotic selection pressure in the community. The relationship between resistance and individual consumption of antimicrobials has also been reported in a cross-sectional study [34], a non-randomized observational study [36], and a case-controlled study [37]. In these study designs, bias is a matter of concern. The impact of macrolide therapy on pharyngeal carriage of macrolide-resistant oral streptococci was investigated in a randomized, double-blind, placebo-controlled study in which healthy volunteers were treated with azithromycin and clarithromycin [38], but no such studies have been performed for *S. pneumoniae*. Once resistant clones become predominant in a community and account for most of the circulating isolates, healthy children most likely acquire resistant isolates even without taking antimicrobials. This process may occur in daycare centers, as shown in the current study. Indeed, Bartoloni et al. reported the dissemination of antimicrobial-resistant commensal *Escherichia coli* without heavy exposure to antimicrobials in a remote Bolivian area [39].

Our data demonstrated that a specific serotype (19F) was primarily responsible for non-susceptibility. The disproportionate distribution of serotypes among non-susceptible isolates was consistent with previous observations of the emergence of globally distributed resistant clones [40–42]. Spain^23F^-1, Taiwan^19F^-14 and their related strains have been described in Vietnam [7, 9]. Further molecular analysis using multi-locus sequence typing (MLST) [43] will demonstrate the presence of globally circulating resistant clones. Molecular analysis will also be a clue to the mystery: why were *S. pneumoniae* strains non-susceptible to carbapenems circulating in Vietnam without the overuse of carbapenems. In a small-scale hospital-based study conducted at the study site in 2011, third generation cephalosporins were frequently used for pneumonia, whereas oral second-generation cephalosporins and penicillins were prescribed for milder cases; carbapenems were not selected at all (Toizumi M, et al. unpublished data).

One solution for the high rate of antimicrobial resistance is the introduction of vaccines. The proportion of PCV-13 serotypes among the carriage isolates was greater than 70% for both the healthy children and the ARI cases, similar to reports from developed countries in the pre-PCV era [14, 44]. Furthermore, PCV13 vaccine serotypes accounted for 90% of the pneumococcal isolates non-susceptible to multiple beta-lactam antimicrobials. Introduction of a vaccine is expected to increase the susceptibility of the circulating strains. Genotypic analysis revealed that *gPSSP* expression accounted for less than 3% of the pneumococcal isolates and that 97.6% of the isolates possessed at least one resistant *pbp* gene. Relatively drug-susceptible pneumococci with non-vaccine serotypes may exchange resistance genes through genetic recombination and acquire higher levels of resistance unless appropriate regulation of antimicrobials (reduction of the area of consumption of antimicrobials) is implemented. In a surveillance study of healthcare utilization conducted in the study area in 2006, approximately 40% of 1355 caregivers of ARI children visited a pharmacy for their first choice of healthcare provider and slightly less than 60% of them purchased antimicrobials (Toizumi M, et al. unpublished data). PCV-13 is expected to reduce the non-susceptible *S. pneumoniae* carriage to a greater extent than PCV-7 [15]. However, careful monitoring of emerging mutants (serotype switching or acquisition of drug resistance genes) is required [45, 46].

We did not detect a clinical impact of the carriage of pneumococcal isolates non-susceptible to multiple beta-lactam antimicrobials. The detection of these non-susceptible pneumococcal isolates in the ARI cases did not lead to significant prolongation of hospitalization. One possible reason was that our study population did not include severe cases who were admitted to the pediatric intensive care unit. Feikin et al. described the positive association between mortality from invasive pneumococcal pneumonia and PEN MICs ≥4 μg/ml (Odds Ratio: 7.1) or cefotaxime MICs ≥2 μg/ml (Odds Ratio: 5.9) after the first 4 hospital days [47].

The current study has some limitations. Information on sibling health conditions was not obtained from the questionnaires; therefore, whether the siblings of the healthy children took antimicrobials during the study period and whether intra-familial transmission occurred were unknown. A previous study in isolated, rural Utah communities demonstrated an association between the detection of non-susceptible *S. pneumoniae* and having a sibling colonized with resistant pneumococci as evidence of intra-familial transmission based on pulsed field gel electrophoresis [48].

Our method of pneumococcal isolation (based upon colony morphology on a non-selective medium) and identification (optochin susceptibility and a bile solubility test) could underestimate NT pneumococcal carriage status because of their morphological differences (less conspicuous dimples of the colonies) from serotypeable *S. pneumoniae*. We considered only bile soluble isolates as pneumococci, which may also lead to the underestimation of NT pneumococcal carriage. Some other members of the viridans group of streptococci might be optochin susceptible which lead to false positive results; to avoid the problem, we defined *S. pneumoniae* carriage as a nasopharyngeal sample was culture positive for *S. pneumoniae* and *lytA* positive, or culture positive for *S. pneumoniae* and *cpsA* positive.

The agar dilution method used for MIC testing may not be the standard method used in many laboratories. However, we found good concordance between the agar dilution method and the microdilution method. Therefore, we believe that the significant increase in non-susceptibility to PEN and other beta-lactam antimicrobials in the present study is a reliable finding.

In Vietnam, the PCV has not been introduced yet and irrational use of antimicrobials is a great issue. Thus data from this study will give us background information to compare with the ongoing study on current situation of antibiotic resistance pattern, serotype predominance, and sequence type distribution of pneumococci in Vietnam.

## Conclusion

*S. pneumoniae* serotype 19F isolates non-susceptible to multiple beta-lactam antimicrobials are widely circulating among children living in central Vietnam. The introduction of a PCV is expected to improve antimicrobial susceptibility.

## Additional files

Additional file 1: **Figure S1.** The epidemiologic-clinical-laboratory form used in the study. (DOCX 276 kb)

Additional file 2: **Figure S2.** Pneumococcal carriage rate by age group. (DOCX 31 kb)

Additional file 3: **Table S1.** MIC Distribution against Pneumococcal Isolates from Study Participants. (DOCX 16 kb)

Additional file 4: **Table S2.** Non-Susceptibility of Penicillin-Susceptible, -Intermediate, and -Resistant *S. pneumoniae* to Other Antimicrobials. (DOCX 16 kb)

Additional file 5: **Figure S3.** Serotype distribution among pneumococcal carriage isolates from healthy children and ARI cases. (DOCX 26 kb)

Additional file 6: **Table S3.** Multiple Beta-Lactam Non-Susceptible Isolates by Serotype/Serogroup. (DOCX 16 kb)

Additional file 7: **Table S4.** *pbp* Genotypes. (DOCX 15 kb)

Additional file 8: **Table S5.** Macrolide Resistance Genes. (DOCX 15 kb)

Additional file 9: Data set used for analysis. (XLSX 98 kb)

## Abbreviations

ARI: Acute respiratory infection
CLSI: Clinical and Laboratory Standards Institute
CTX: Cefotaxime
gPRSP: genotypic penicillin-resistant *S. pneumoniae*
gPSSP: genotypic penicillin-sensitive *S. pneumoniae*
IPD: Invasive pneumococcal disease
MEM: Meropenem
MIC: Minimum inhibitory concentration
MLST: multi-locus sequence typing
PCR: Polymerase chain reaction
PCV: Pneumococcal conjugate vaccine
PEN: Penicillin
PRSP: penicillin-resistant *Streptococcus pneumoniae*
