# Supplementary figures and images for: An alarmingly high nasal carriage rate of Streptococcus pneumoniae serotype 19F non-susceptible to multiple beta-lactam antimicrobials among Vietnamese children
Source: BMC Infect Dis. 2019 Mar 11;19:241. doi: 10.1186/s12879-019-3861-2 (PMC6416861; doi:10.1186/s12879-019-3861-2)

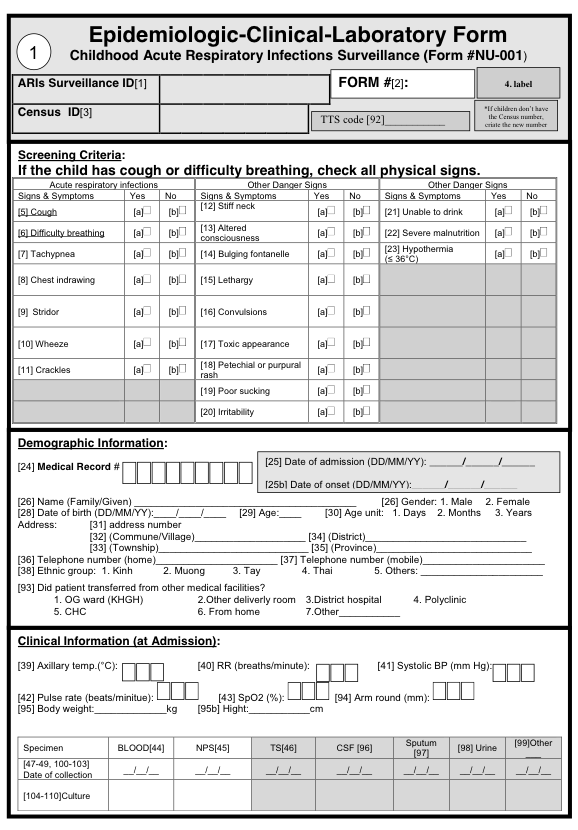


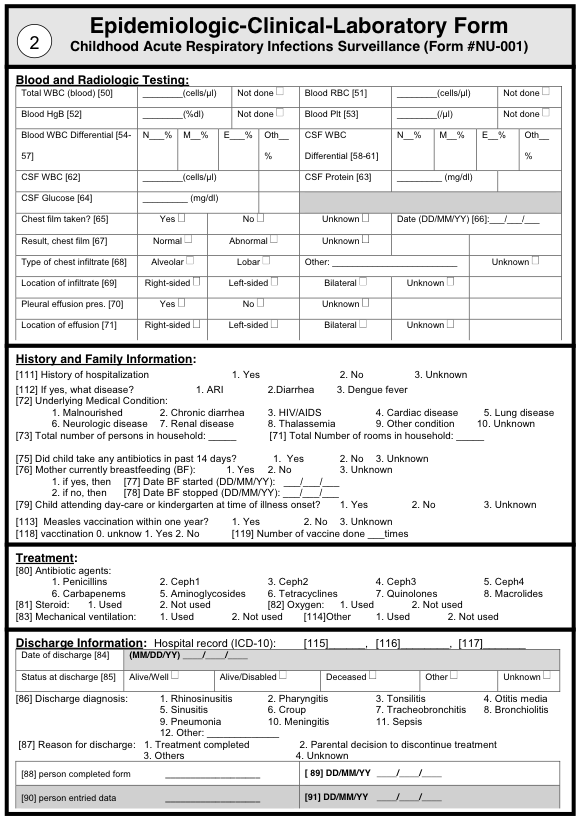


**Figure S1.** The epidemiologic-clinical-laboratory form used in the study.

Supplement: Supplementary file 1 — Figure S1. The epidemiologic-clinical-laboratory form used in the study. (DOCX 276 kb) [file 12879_2019_3861_MOESM1_ESM.docx]

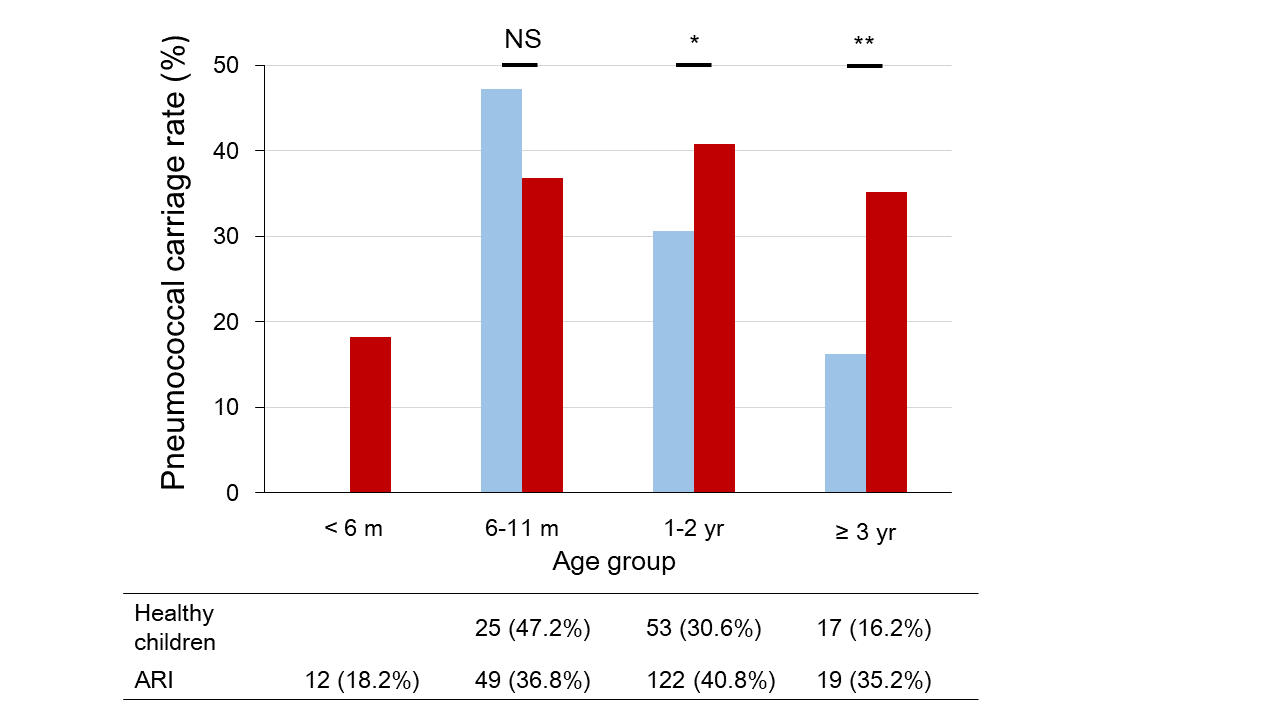


**Figure S2.** Pneumococcal carriage rate by age group.

*:p=0.0276/**:p=0.0067 (analyzed using the Chi-square test)

Supplement: Supplementary file 2 — Figure S2. Pneumococcal carriage rate by age group. (DOCX 31 kb) [file 12879_2019_3861_MOESM2_ESM.docx]
