# Supplementary material for: An alarmingly high nasal carriage rate of Streptococcus pneumoniae serotype 19F non-susceptible to multiple beta-lactam antimicrobials among Vietnamese children
Source: BMC Infect Dis. 2019 Mar 11;19:241. doi: 10.1186/s12879-019-3861-2 (PMC6416861; doi:10.1186/s12879-019-3861-2)
Supplement: Supplementary file 3 — Table S1. MIC Distribution against Pneumococcal Isolates from Study Participants. (DOCX 16 kb) [file 12879_2019_3861_MOESM3_ESM.docx]

**Table S1**. MIC Distribution against Pneumococcal Isolates from Study Participants

|  | Range | Healthy children | | ARI | | Total | |
| --- | --- | --- | --- | --- | --- | --- | --- |
|  |  | MIC50 | MIC90 | MIC50 | MIC90 | MIC50 | MIC90 |
| Penicillin (n = 283) | 0.016- ≥ 16 | 1 | 4 | 2 | 4 | 1 | 4 |
| Amoxicillin (n = 283) | 0.016- ≥ 16 | 1 | 8 | 1 | 8 | 1 | 8 |
| Ampicillin (n = 283) | 0.016- ≥ 32 | 2 | 8 | 2 | 8 | 2 | 8 |
| Amoxicillin/clavulanate (n = 283) | 0.016-8 | 0.5 | 4 | 1 | 4 | 1 | 4 |
| Cefaclor (n = 283) | 0.5- > 128 | 16 | > 128 | 64 | > 128 | 64 | > 128 |
| Cefuroxime (n = 283) | 0.016-64 | 4 | 32 | 4 | 16 | 4 | 16 |
| Cefotaxime (n = 283) | 0.008-16 | 1 | 8 | 1 | 2 | 1 | 2 |
| Cefepime (n = 283) | 0.032-8 | 1 | 2 | 1 | 2 | 1 | 2 |
| Imipenem (n = 283) | 0.008-8 | 0.25 | 1 | 0.25 | 0.5 | 0.25 | 1 |
| Meropenem (n = 283) | 0.016-4 | 0.5 | 1 | 0.5 | 1 | 0.5 | 1 |
| Erythromycin (n = 283) | 0.032- > 128 | >128 | >128 | 2 | > 128 | 128 | > 128 |
| Azithromycin (n = 283) | 0.063- > 128 | >128 | >128 | 8 | > 128 | > 128 | > 128 |
| Clarithromycin (n = 283) | 0.032- > 128 | >128 | >128 | 2 | > 128 | > 128 | > 128 |
| Chloramphenicol (n = 283) | 1-16 | 8 | 16 | 4 | 16 | 4 | 16 |
| Trimethoprim/sulfamethoxazole (n = 283) | 4- > 128 | 128 | >128 | 128 | > 128 | 128 | > 128 |
| Clindamycin (n = 283) | 0.016- > 128 | >128 | >128 | 128 | > 128 | 128 | > 128 |
| Ofloxacin (n = 283) | 0.5-32 | 2 | 4 | 2 | 4 | 2 | 4 |
| Vancomycin (n = 283) | 0.125-1 | 0.5 | 1 | 0.25 | 0.5 | 0.5 | 0.5 |
| Tetracycline (n = 283) | 0.125-128 | 32 | 64 | 32 | 64 | 32 | 64 |
| Rifampicin (n = 283) | 0.016- ≥ 2 | 0.125 | 0.25 | 0.25 | 0.5 | 0.125 | 0.25 |
