# Supplementary material for: An alarmingly high nasal carriage rate of Streptococcus pneumoniae serotype 19F non-susceptible to multiple beta-lactam antimicrobials among Vietnamese children
Source: BMC Infect Dis. 2019 Mar 11;19:241. doi: 10.1186/s12879-019-3861-2 (PMC6416861; doi:10.1186/s12879-019-3861-2)
Supplement: Supplementary file 4 — Table S2. Non-Susceptibility of Penicillin-Susceptible, -Intermediate, and -Resistant S. pneumoniae to Other Antimicrobials. (DOCX 16 kb) [file 12879_2019_3861_MOESM4_ESM.docx]

**Table S2**. Non-Susceptibility of Penicillin-Susceptible, -Intermediate, and -Resistant *S. pneumoniae* to Other Antimicrobials

|  | PEN MIC ≤ 2 μg/ml  (n = 242) | PEN MIC = 4 μg/ml  (n = 43) | PEN MIC ≥ 8 μg/ml  (n = 10) |
| --- | --- | --- | --- |
| Amoxicillin (nonmeningitis) | 34 (14.0%) | 25 (58.1%) | 10 (100%) |
| Amoxicillin-clavulanic acid (nonmeningitis) | 12 (5.0%) | 17 (39.5%) | 8 (80.0%) |
| Cefaclor | 207 (85.5%) | 41 (95.3%) | 9 (90.0%) |
| Cefuroxime (oral) | 152 (62.8%) | 43 (100%) | 10 (100%) |
| Cefotaxime (nonmeningitis) | 36 (14.9%) | 32 (74.4%) | 8 (80.0%) |
| Cefotaxime (meningitis) | 105 (43.4%) | 43 (100%) | 8 (80.0%) |
| Cefepime (nonmeningitis) | 32 (13.2%) | 36 (83.7%) | 7 (70.0%) |
| Cefepime (meningitis) | 143 (59.1%) | 43 (100%) | 9 (90.0%) |
| Imipenem | 157 (64.9%) | 43 (100%) | 10 (100%) |
| Meropenem | 170 (70.2%) | 43 (100%) | 10 (100%) |
| Erythromycin | 204 (84.3%) | 43 (100%) | 10 (100%) |
| Azithromycin | 210 (86.8%) | 43 (100%) | 10 (100%) |
| Clarithromycin | 201 (83.1%) | 43 (100%) | 10 (100%) |
| Clindamycin | 190 (78.5%) | 43 (100%) | 10 (100%) |
| Tetracycline | 213 (88.0%) | 40 (93.0%) | 10 (100%) |
| Chloramphenicol | 97 (40.1%) | 28 (65.1%) | 4 (40.0%) |
| Trimethoprim-sulfamethoxazole | 218 (90.1%) | 42 (97.7%) | 9 (90.0%) |
| Ofloxacin | 52 (21.5%) | 14 (32.6%) | 3 (30.0%) |
| Rifampicin | 2 (0.8%) | 0 (0%) | 0 (0%) |
| Vancomycin | 0 (0%) | 0 (0%) | 0 (0%) |

Results for susceptibility testing of 295 isolates.
