# Supplementary material for: An alarmingly high nasal carriage rate of Streptococcus pneumoniae serotype 19F non-susceptible to multiple beta-lactam antimicrobials among Vietnamese children
Source: BMC Infect Dis. 2019 Mar 11;19:241. doi: 10.1186/s12879-019-3861-2 (PMC6416861; doi:10.1186/s12879-019-3861-2)
Supplement: Supplementary file 5 — Figure S3. Serotype distribution among pneumococcal carriage isolates from healthy children and ARI cases. (DOCX 26 kb) [file 12879_2019_3861_MOESM5_ESM.docx]

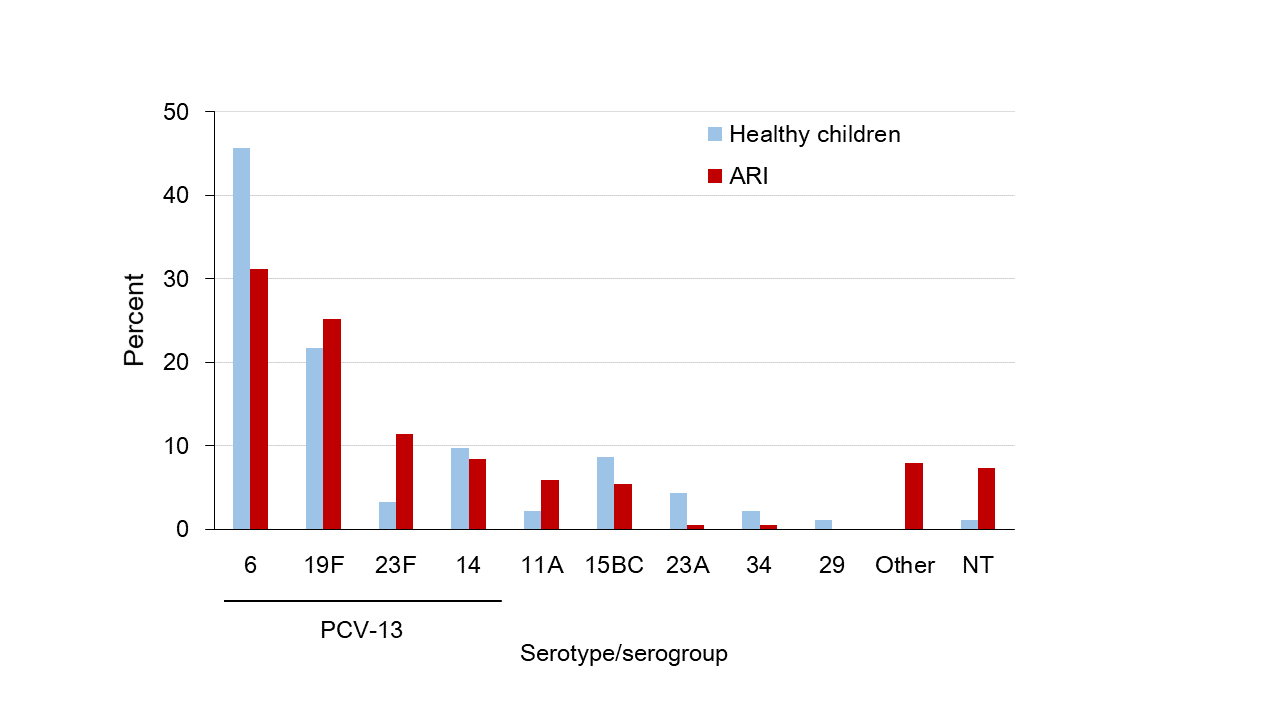


**Figure S3.** Serotype distribution among pneumococcal carriage isolates from healthy children (n=92) and ARI cases (n=202).
