# Supplementary material for: An alarmingly high nasal carriage rate of Streptococcus pneumoniae serotype 19F non-susceptible to multiple beta-lactam antimicrobials among Vietnamese children
Source: BMC Infect Dis. 2019 Mar 11;19:241. doi: 10.1186/s12879-019-3861-2 (PMC6416861; doi:10.1186/s12879-019-3861-2)
Supplement: Supplementary file 6 — Table S3. Multiple Beta-Lactam Non-Susceptible Isolates by Serotype/Serogroup. (DOCX 16 kb) [file 12879_2019_3861_MOESM6_ESM.docx]

**Table S3**. Multiple Beta-Lactam Non-Susceptible* Isolates by Serotype/Serogroup

| Serotype/ Serogroup | Multiple beta-lactam non-susceptible isolates (n = 40) | Other  pneumococcal isolates (n = 245) | Odds ratio (95% CI) | P |
| --- | --- | --- | --- | --- |
| 19F (n = 67) | 20 (29.9%) | 47 | 4.23 (2.11-8.50) | < 0.0001 |
| 6 (n = 100) | 14 (14.0%) | 86 | 1.00 (0.50-2.02) | 0.9960 |
| 23F (n = 24) | 2 (8.3%) | 22 | 0.54 (0.12-2.37) | 0.4042 |
| 14 (n = 25) | 0 | 25 | - | - |
| 11A (n = 12) | 1 (8.3%) | 11 | 0.55 (0.07-4.36) | 0.5641 |
| 15BC (n = 18) | 0 | 18 | - | - |
| Other^**^ (n = 23) | 3 (13.0%) | 20 | 0.91 (0.26-3.22) | 0.8865 |
| NT (n = 16) | 0 | 16 | - | - |

This analysis was performed for cases with pneumococcal isolates with a single serotype (n = 285). ^**^: includes serotypes 23A, 29, and 34

*PEN MIC ≥ 4 μg/mL, CTX MIC ≥ 2 μg/mL and MEM MIC ≥ 0.5 μg/mL
