# Supplementary material for: An alarmingly high nasal carriage rate of Streptococcus pneumoniae serotype 19F non-susceptible to multiple beta-lactam antimicrobials among Vietnamese children
Source: BMC Infect Dis. 2019 Mar 11;19:241. doi: 10.1186/s12879-019-3861-2 (PMC6416861; doi:10.1186/s12879-019-3861-2)
Supplement: Supplementary file 7 — Table S4. pbp Genotypes. (DOCX 15 kb) [file 12879_2019_3861_MOESM7_ESM.docx]

**Table S4**. *pbp* Genotypes

|  | n (%) | Healthy children  (n = 89) | ARI cases  (n = 201) |
| --- | --- | --- | --- |
| *gPSSP* | 7 (2.4%) | 6 | 1 |
| *gPISP* |  |  |  |
| *pbp1a* | 1 (0.3%) | 1 | 0 |
| *pbp2b* | 0 | 0 | 0 |
| *pbp2x* | 1 (0.3%) | 0 | 1 |
| *pbp1a+pbp2x* | 3 (1.0%) | 2 | 1 |
| *pbp1a+pbp2b* | 1 (0.3%) | 0 | 1 |
| *pbp2b+pbp2x* | 12 (4.1%) | 4 | 8 |
| *gPRSP* |  |  |  |
| *pbp1a+pbp2b+pbp2x* | 265 (91.4%) | 76 | 189 |
