# Supplementary material for: An alarmingly high nasal carriage rate of Streptococcus pneumoniae serotype 19F non-susceptible to multiple beta-lactam antimicrobials among Vietnamese children
Source: BMC Infect Dis. 2019 Mar 11;19:241. doi: 10.1186/s12879-019-3861-2 (PMC6416861; doi:10.1186/s12879-019-3861-2)
Supplement: Supplementary file 8 — Table S5. Macrolide Resistance Genes. (DOCX 15 kb) [file 12879_2019_3861_MOESM8_ESM.docx]

**Table S5**. Macrolide Resistance Genes

|  | n (%) | Healthy children  (n = 89) | ARI cases  (n = 201) |
| --- | --- | --- | --- |
| *ermB+ mefA+* | 70 (24.1%) | 14 | 56 |
| *ermB+ mefA-* | 192 (66.2%) | 65 | 127 |
| *ermB- mefA+* | 20 (6.9%) | 6 | 14 |
| *ermB- mefA-* | 8 (2.8%) | 4 | 4 |
